# Supplementary figures and images for: A novel gene signature related to oxidative stress predicts the prognosis in clear cell renal cell carcinoma
Source: PeerJ. 2023 Feb 8;11:e14784. doi: 10.7717/peerj.14784 (PMC9921988; doi:10.7717/peerj.14784)

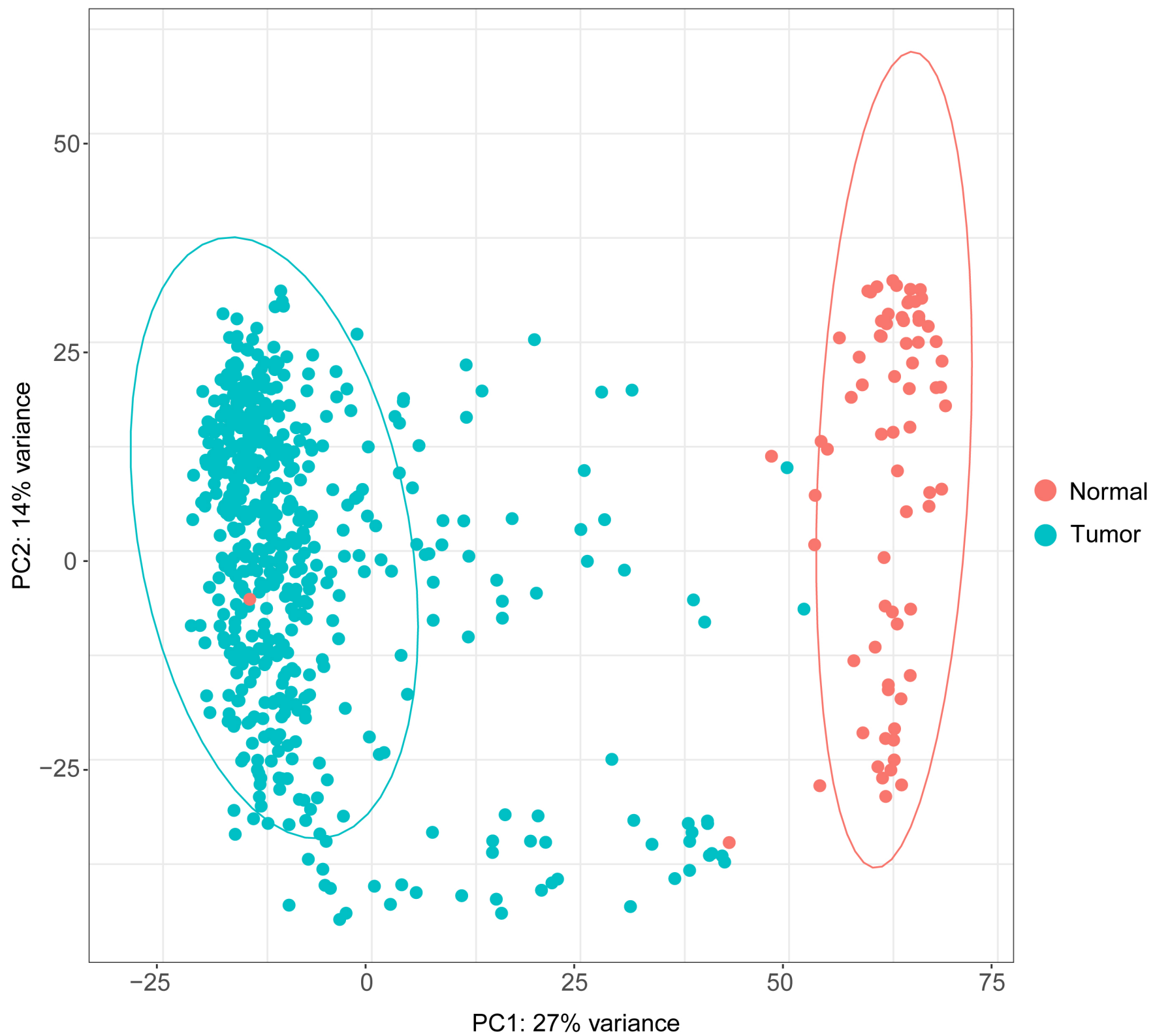

Supplement: Supplemental Information 1 [file peerj-11-14784-s001.pdf]

**A**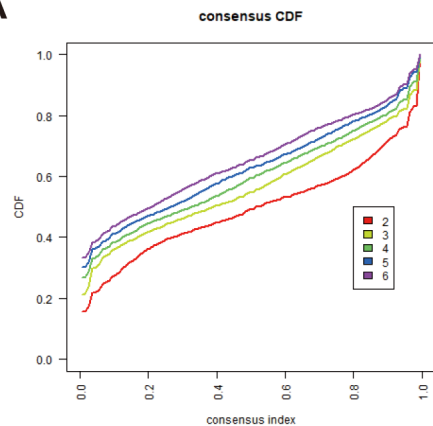**B**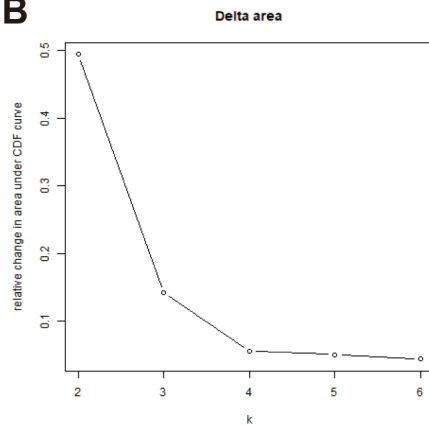**C**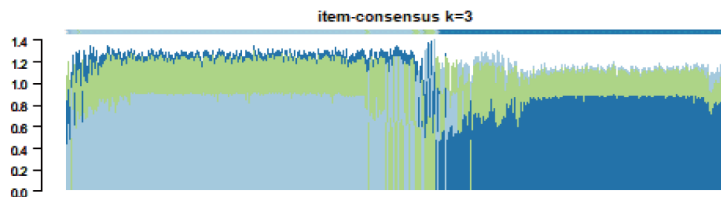**D**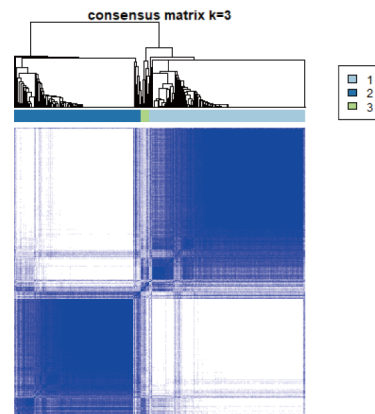

Supplement: Supplemental Information 2 — (A) Cumulative distribution function (CDF) curve. (B) Delta area curve, presenting the change of area under the CDF curve. (C) Item-Consensus Plot for K = 3. each bar chart represents a sample to illustrate its purity. (D) The matrix heatmap for K = 3. The value of consistency matrix from 0 to 1 represents the color from white to blue. [file peerj-11-14784-s002.pdf]

**A**

pathologic\_T

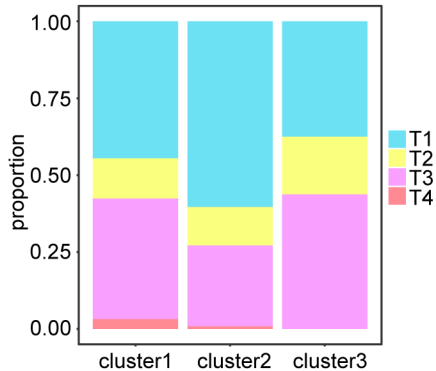**B**

pathologic\_M

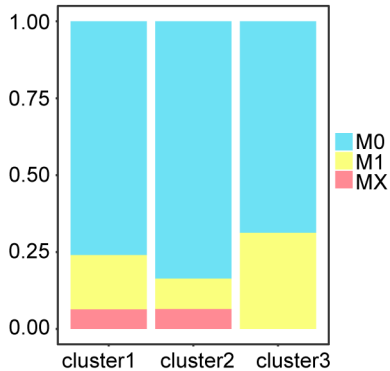**C**

pathologic\_N

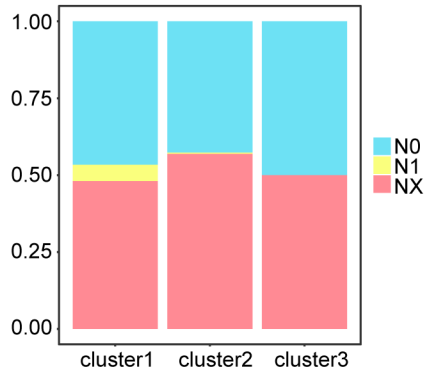

Supplement: Supplemental Information 3 [file peerj-11-14784-s003.pdf]

A

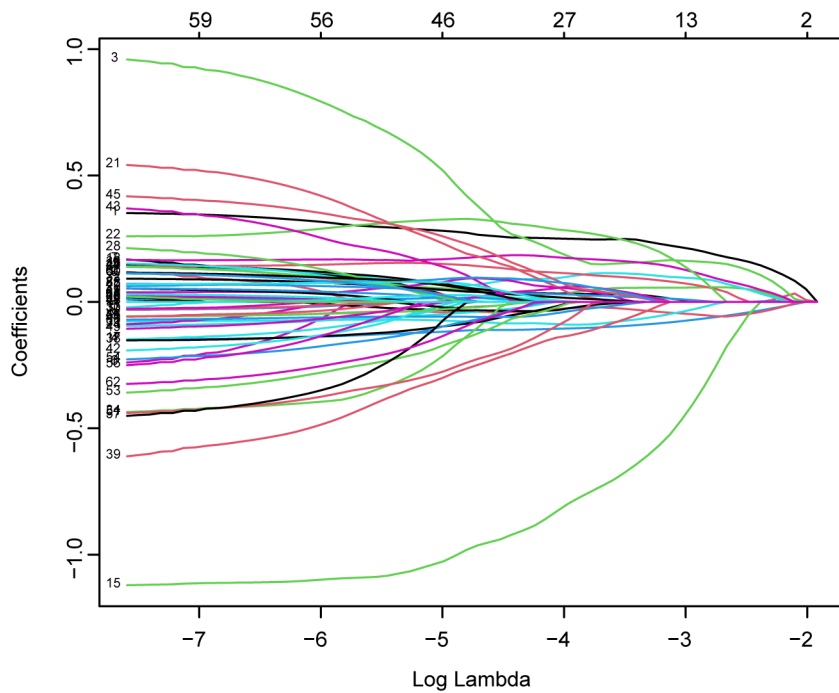

B

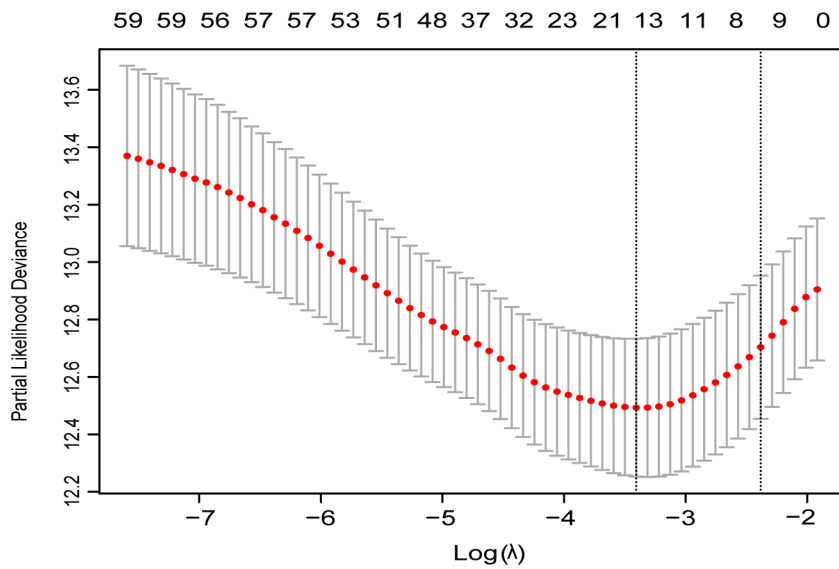

Supplement: Supplemental Information 4 — (A, B) genes selected through Lasso regression. [file peerj-11-14784-s004.pdf]

A

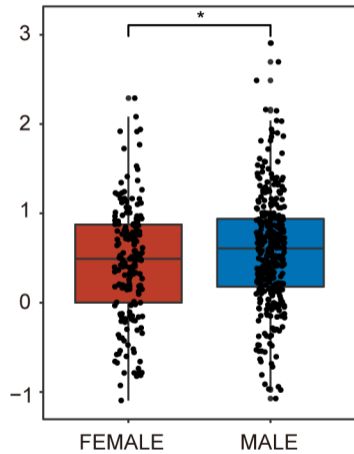

B

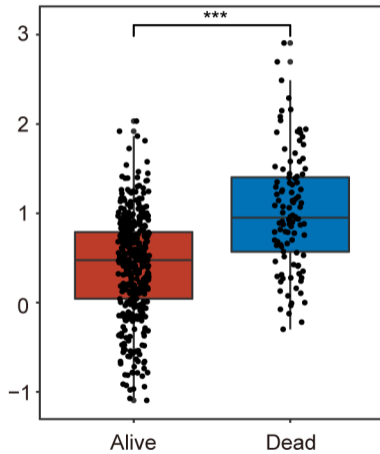

C

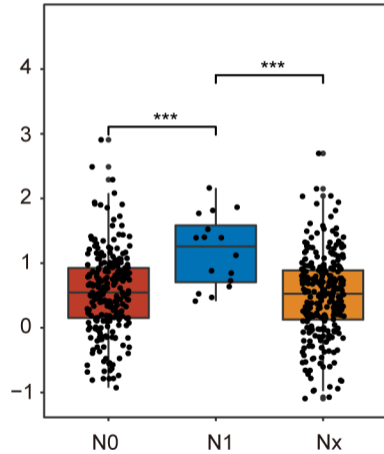

Supplement: Supplemental Information 5 — (A) Different risk scores between male and female in ccRCC patients (B) Patients with different DSS outcomes have different risk scores. (C) Patients with lymph node metastasis are associated with higher risk scores. *: P < 0.05, **: P < 0.01, ***: P < 0.001, ****: P < 0.0001. [file peerj-11-14784-s005.pdf]
